# O Estilo de Vida dos Muito Idosos Importa

**DOI:** 10.36660/abc.20200586

**Published:** 2020-11-01

**Affiliations:** 1 Hospital Israelita Albert Einstein São PauloSP Brasil Hospital Israelita Albert Einstein, São Paulo, SP – Brasil

**Keywords:** Envelhecimento, Apolipoproteínas E, Estilo de Vida

De acordo com as estimativas e projeções do relatório “Perspectivas da População Mundial: Revisão de 2019”, a expectativa de vida continuará a aumentar nos próximos 30 anos em todo o mundo, a porcentagem da população com 65 anos ou mais saltará de 9% em 2020 para 16% em 2050, e o número de pessoas com 80 anos ou mais triplicará até 2050.^1^

A biologia do envelhecimento e dos genes envolvidos vem sendo estudada há muito tempo como base para a busca de terapias que possam mitigar processos relacionados ao envelhecimento, como demência e doenças cardiovasculares. Entre os múltiplos aspectos explorados, a pesquisa envolvendo a apolipoproteína E (apoE) assume particular relevância. A apoE é uma proteína de 299 aminoácidos sintetizada principalmente pelos hepatócitos. Como qualquer outra apolipoproteína, a apoE é um constituinte das lipoproteínas e, portanto, tem um papel no metabolismo lipídico. No plasma, a apoE é transportada principalmente por lipoproteínas ricas em triglicerídeos e funciona como mediadora da depuração de seus remanescentes pelo receptor da lipoproteína de baixa densidade. No cérebro, a apoE produzida *in situ* atua na redistribuição de lipídios para os neurônios, bem como na depuração de β-amiloide, muito conhecido por sua associação com a doença de Alzheimer.^2^^,^^3^

Sabe-se que polimorfismos no gene APOE estão associados a processos patológicos. Três alelos comuns da APOE são descritos: o ϵ2 (o menos frequente), ϵ3 (o mais prevalente e considerado como o tipo selvagem ou “neutro”) e ϵ4. Com base nesses alelos, um indivíduo específico pode exibir um genótipo homozigoto (APOE ϵ2/ϵ2, APOE ϵ3/ϵ3 ou APOE ϵ4/ϵ4) ou heterozigoto (APOE ϵ3/ϵ2, APOE ϵ4/ϵ2 ou APOE ϵ4/ϵ3). A apoE2 e apoE4 diferem da apoE3 por uma única substituição de aminoácido. No entanto, esses polimorfismos modificam significativamente a estrutura e função da apoE.^2^^,^^3^

A apoE é o principal fator genético que predispõe à doença de Alzheimer de início tardio. Em comparação com a apoE3, a presença da apoE4 confere um maior risco de doença de Alzheimer, enquanto a apoE2 está associada a um risco menor. Grandes estudos de coorte também mostraram uma associação entre a apoE4 e um maior risco de doença cardiovascular aterosclerótica, mortalidade cardiovascular e mortalidade por todas as causas.^2^^,^^3^

Nesse contexto, este número dos Arquivos Brasileiros de Cardiologia publica um artigo da coorte prospectiva de Veranópolis, investigando a relação entre o genótipo da APOE e a mortalidade em uma população pouco estudada, composta por indivíduos com 80 anos ou mais.^4^ Em um período muito longo de seguimento (de até 21 anos), os autores não detectaram diferença no risco de mortalidade entre os indivíduos APOE ϵ3/ϵ3 em comparação com os participantes com APOE ϵ3/ϵ4.

O achado “negativo” foi possivelmente consequência da amostra altamente seletiva e do pequeno número de participantes (16 com o genótipo APOE ϵ3/ϵ4 e 53 com o genótipo APOE ϵ3/ϵ3), o qual acarretou uma falta de poder do estudo para detectar pequenas diferenças. Em uma grande coorte da população geral dinamarquesa, apenas uma mínima diferença na mediana da sobrevida associada a diferentes genótipos foi relatada (86,4 anos em portadores de ɛ33 e 85,9 anos em portadores de ɛ43).^5^ Além disso, entre os indivíduos de Veranópolis, ninguém tinha o genótipo APOE ϵ4/ϵ4, que está associado à maior mortalidade.^5^

Por outro lado, mesmo com o número limitado de participantes, os autores foram capazes de detectar uma associação entre fatores de risco tradicionais e mortalidade geral. Essa foi a descoberta mais surpreendente do estudo. As análises ajustadas demonstraram efeitos deletérios claros do tabagismo e diabetes mellitus, e um poderoso efeito protetor da atividade física.^4^

Os resultados também apontam para uma relação inversa entre pressão arterial e mortalidade: cada aumento de 1 mmHg na pressão arterial sistólica foi associado a uma redução de 2% no risco de morte.^4^ Esse achado parece intrigante, uma vez que ensaios clínicos randomizados e controlados apoiam a redução intensiva da pressão arterial em pessoas idosas para prevenir eventos duros.^6^ A presença de fatores de confusão pode ser responsável por este achado, embora um efeito genuinamente prejudicial da redução excessiva da pressão arterial nesta população também seja possível.

O aumento do risco de morte associado ao diabetes mellitus no estudo de Veranópolis, embora não seja uma surpresa, destaca a importância de medidas voltadas para a prevenção da doença. Nesse sentido, intervenções no estilo de vida com restrição calórica na dieta e atividade física, bem como algumas opções farmacológicas (por exemplo, metformina), têm demonstrado prevenir ou retardar o desenvolvimento do diabetes.^7^

Os efeitos nocivos do tabagismo no estudo de Veranópolis são consistentes com outros relatos. Uma grande meta-análise de indivíduos com 60 anos ou mais de estudos de coorte prospectivos mostrou inequivocamente que o tabagismo é um forte fator de risco independente de eventos cardiovasculares, avançando a mortalidade cardiovascular em mais de cinco anos.^8^ Além disso, alguns anos de cessação do tabagismo foram suficientes para detectar um efeito benéfico na mortalidade cardiovascular, e esse benefício aumentou ao longo do tempo após a cessação.^8^

O estudo de Veranópolis mostrou que os idosos que se exercitam (gasto energético semanal de pelo menos 4.000 kcal) apresentam uma impressionante redução de 51% no risco de morte. Esse resultado é corroborado por várias outras publicações.^9^^–^^13^ Mesmo a atividade física de intensidade leve reduz a mortalidade em idosos. Em um estudo prospectivo da Espanha com um período de acompanhamento de 13 anos, os participantes (com 65 anos ou mais) no nível de atividade física de maior intensidade apresentaram o menor risco de mortalidade após ajuste para várias covariáveis. Aqueles com atividade física de intensidade leve tiveram um prognóstico melhor do que indivíduos sedentários.^10^ Da mesma forma, dados da *Women's Health Initiative* mostraram que mesmo a atividade física de intensidade leve reduz eventos cardiovasculares e mortalidade por todas as causas em mulheres com média de idade de 79 anos.^11^^,^^12^ As diretrizes recomendam atividade física com múltiplos componentes para idosos, combinando exercícios aeróbicos, de fortalecimento muscular e de equilíbrio, para promover benefícios abrangentes à saúde, incluindo a prevenção de quedas. Se o indivíduo idoso não puder fazer atividade aeróbica de intensidade moderada devido a uma doença crônica, ele deve ser incentivado a ser tão fisicamente ativo quanto permitido por sua condição.^14^

As análises de dados observacionais sofrem com a possibilidade de viés introduzido pela falta de ajustes adequados e fatores de confusão desconhecidos, bem como causalidade reversa. Os indivíduos sedentários são mais propensos a desfechos adversos devido à falta dos efeitos benéficos da atividade física ou porque já apresentam condições mórbidas que os impedem de se exercitar? Não obstante, considerando os múltiplos estudos com ajustes apropriados para comorbidades e estado funcional, as evidências apontam para uma mensagem clara: o estilo de vida faz a diferença, mesmo em pessoas muito idosas.

Dito isso, as implicações para a comunidade médica e o médico assistente são diretas: a idade não deve ser uma barreira para a implementação de modificações no estilo de vida. Ao contrário, esforços devem ser feitos para criar e facilitar o acesso dos idosos a programas voltados para o estilo de vida, incluindo aqueles relacionados à cessação do tabagismo, atividade física, hábitos alimentares e bem-estar psicológico. Diretrizes podem ser desenvolvidas. Estratégias para envolver os idosos em atividades saudáveis devem ser estudadas. Novas tecnologias, como o uso de acelerômetro^11^^,^^12^ e plataformas de internet,^15^ podem ser utilizadas para otimizar os resultados. Os cuidadores e demais profissionais devem ser treinados para orientar a atividade física, reconhecendo os limites e peculiaridades desse subgrupo específico. Com ações multifacetadas, a modificação do estilo de vida pode ser um pilar essencial para reduzir a carga de doenças relacionadas ao envelhecimento nas próximas décadas.
