# Supplementary material for: O Estilo de Vida dos Muito Idosos Importa
Source: Arq Bras Cardiol. 2020 Nov 1;115(5):882–4. [Article in Portuguese] doi: 10.36660/abc.20200586 (PMC8452215; doi:10.36660/abc.20200586)
Supplement: Translation

# Translation: Lifestyle in the Very Elderly Matters

**Affiliations:** 1 São PauloSP Brazil Hospital Israelita Albert Einstein, São Paulo, SP – Brazil

**Keywords:** Aging, Apolipoproteins E, Life Style

According to the estimates and projections of the 2019 Revision of World Population Prospects, life expectancy will continue to rise in the following 30 years worldwide, the percentage of the population aged 65 years or over will jump from 9% in 2020 to 16% in 2050, and the number of persons aged 80 years or over will triple up to 2050.^1^

The biology of aging and implicated genes has been studied for a long time as a basis for the search for therapies that may mitigate age-related processes, such as dementia and cardiovascular disease. Among the multitude of aspects that have been explored, the research involving apolipoprotein E (apoE) assumes particular relevance. ApoE is a 299-amino acid protein mainly synthesized by the hepatocytes. Like any other apolipoprotein, apoE is a constituent of lipoproteins and, therefore, has a role in lipid metabolism. In the plasma, apoE is primarily carried by triglyceride-rich lipoproteins and mediates the clearance of their remnants by the low-density lipoprotein receptor. In the brain, *in situ* produced apoE acts on the redistribution of lipids to neurons, as well as in the clearance of β-amyloid, much known for its association with Alzheimer disease.^2^^,^^3^

It is well acknowledged that polymorphisms in the APOE gene are associated with pathological processes. Three common APOE alleles are described: the ϵ2 (the least frequent), ϵ3 (the most prevalent and considered to be the wild or “neutral” type), and ϵ4. Based on these alleles, a specific individual can display a homozygous (APOE ϵ2/ϵ2, APOE ϵ3/ϵ3, or APOE ϵ4/ϵ4) or a heterozygous (APOE ϵ3/ϵ2, APOE ϵ4/ϵ2, or APOE ϵ4/ϵ3) genotype. ApoE2 and apoE4 differ from apoE3 by one single amino acid substitution. Nevertheless, these polymorphisms significantly modify the structure and function of apoE.^2^^,^^3^

ApoE is the major genetic factor that predisposes to late-onset Alzheimer disease. Compared to apoE3, the presence of apoE4 confers a higher risk of Alzheimer disease, whereas apoE2 is associated with a lower risk. Large cohort studies also showed an association between apoE4 and a higher risk of atherosclerotic cardiovascular disease, cardiovascular mortality, and all-cause mortality.^2^^,^^3^

In this context, this issue of the *Arquivos Brasileiros de Cardiologia* publishes an article from the Veranópolis prospective cohort, investigating the relationship between APOE genotype and mortality in a population not commonly studied, composed by individuals aged 80 years or older.^4^ In a very long follow-up (up to 21 years), the authors did not detect a difference in the hazard of mortality between APOE ϵ3/ϵ3 individuals, as compared with APOE ϵ3/ϵ4 participants.

The “negative” finding was possibly a consequence of the highly selective sample and the small number of participants (16 with the APOE ϵ3/ϵ4 genotype and 53 with the APOE ϵ3/ϵ3 genotype), which imposed a lack of power to detect small differences. In a large cohort of the general Danish population, only a minimal difference in the median survival associated with different genotypes was reported (86.4 years in ɛ33 carriers and 85.9 years in ɛ43 carriers).^5^ Moreover, among the Veranópolis subjects, no one had the APOE ϵ4/ϵ4 genotype, which is associated with the highest mortality.^5^

Conversely, even with the limited number of participants, the authors were able to detect an association between traditional risk factors and overall mortality. That was the most striking finding of the study. Adjusted analyses demonstrated clear deleterious effects of smoking and diabetes mellitus, and a powerful protective effect of physical activity.^4^

The results also point to an inverse relationship between blood pressure and mortality: each 1 mmHg rise in systolic blood pressure was associated with a 2% reduction in the risk of death.^4^ This finding seems intriguing, since randomized controlled trials support intensive blood pressure lowering in older persons to prevent hard events.^6^ The presence of confounders may be responsible for this finding, although a genuinely detrimental effect of excessive blood pressure lowering in this population is also possible.

The increased risk of death associated with diabetes mellitus in the Veranópolis study, although not a surprise, highlights the importance of measures devoted to the prevention of the disease. In this regard, lifestyle interventions with caloric restriction in the diet and physical activity, as well as some pharmacological options (e.g., metformin), have shown to prevent or delay the development of diabetes.^7^

The harmful effects of smoking in the Veranópolis study are consistent with other reports. A large meta-analysis involving subjects aged 60 or older from prospective cohort studies unequivocally showed that smoking is a strong independent risk factor of cardiovascular events, advancing cardiovascular mortality by more than five years.^8^ Moreover, a few years of smoking cessation were enough to detect a beneficial effect on cardiovascular mortality, and this benefit increased over time after cessation.^8^

The Veranópolis study showed that older people who exercised (weekly energy expenditure of at least 4000 kcal) had an impressive 51% lower risk of dying. This result is corroborated by several other publications.^9^^–^^13^ Even light-intensity physical activity has been shown to reduce mortality in older people. In a prospective study from Spain with a follow-up period of 13 years, participants (aged 65 years or older) in the highest-intensity level of physical activity had the lowest mortality risk after adjustment for several covariates. Those with light-intensity physical activity had a better prognosis than sedentary individuals.^10^ Similarly, data from the Women's Health Initiative have shown that even light-intensity physical activity reduces cardiovascular events and all-cause mortality in women with a mean age of 79 years.^11^^,^^12^ Guidelines have recommended multicomponent physical activity for older people, combining aerobic, muscle-strengthening, and balance exercises, to promote comprehensive health benefits, including prevention of falls. If the older adult cannot do moderate-intensity aerobic activity due to a chronic disease, he or she should be encouraged to be as physically active as allowed by his or her condition.^14^

Analyses of observational data suffer from the possibility of bias introduced by lack of adequate adjustments and unknown confounders, as well as reverse causality. Are sedentary individuals more prone to adverse outcomes because of the lack of the beneficial effects of physical activity or because they already have morbid conditions that prevent them from exercising? Nevertheless, considering the multiple studies with appropriate adjustments for comorbidities and functional status, the evidence points to a clear message: lifestyle makes a difference, even in very old persons.

That said, the implications for the medical community and the attending physician are straightforward: age should not be a barrier to the implementation of lifestyle modifications. On the contrary, efforts should be made to create and facilitate the access of older people to programs focused on lifestyle, including those related to smoking cessation, physical activity, dietary habits, and psychological well-being. Guidelines can be developed. Strategies to engage older people in healthy activities should be studied. Novel technologies, such as the use of accelerometer^11^^,^^12^ and internet platforms,^15^ can be used to optimize the results. Caregivers and other personnel should be trained to guide physical activity, recognizing the limits and peculiarities of this specific subgroup. With multifaceted actions, lifestyle modification can be an essential pillar to reduce the burden of age-related diseases in the coming decades.
